# Atg1, a key regulator of autophagy, functions to promote MAPK activation and cell death upon calcium overload in fission yeast

**DOI:** 10.15698/mic2023.06.798

**Published:** 2023-05-10

**Authors:** Teruaki Takasaki, Ryosuke Utsumi, Erika Shimada, Asuka Bamba, Kanako Hagihara, Ryosuke Satoh, Reiko Sugiura

**Affiliations:** 1 Laboratory of Molecular Pharmacogenomics, Department of Pharmaceutical Sciences, Faculty of Pharmacy, Kindai University, Higashi-Osaka, 577-8502, Japan.; 2 Laboratory of Hygienic Science, Department of Pharmacy, Hyogo Medical University, Kobe, 650-8530, Japan.

**Keywords:** Pmk1 MAPK, Autophagy, Cell death, Fission yeast, Calcium tolerance

## Abstract

Autophagy promotes or inhibits cell death depending on the environment and cell type. Our previous findings suggested that Atg1 is genetically involved in the regulation of Pmk1 MAPK in fission yeast. Here, we showed that Δ*atg1* displays lower levels of Pmk1 MAPK phosphorylation than did the wild-type (WT) cells upon treatment with a 1,3-β-D-glucan synthase inhibitor micafungin or CaCl_2_, both of which activate Pmk1. Moreover, the overproduction of Atg1, but not that of the kinase inactivating Atg1^D193A^ activates Pmk1 without any extracellular stimuli, suggesting that Atg1 may promote Pmk1 MAPK signaling activation. Notably, the overproduction of Atg1 induces a toxic effect on the growth of WT cells and the deletion of Pmk1 failed to suppress the cell death induced by Atg1, indicating that the Atg1-mediated cell death requires additional mechanism(s) other than Pmk1 activation. Moreover, *atg1* gene deletion induces tolerance to micafungin and CaCl_2_, whereas *pmk1* deletion induces severe sensitivities to these compounds. The Δ*atg1*Δ*pmk1* double mutants display intermediate sensitivities to these compounds, showing that *atg1* deletion partly suppressed growth inhibition induced by Δ*pmk1*. Thus, Atg1 may act to promote cell death upon micafungin and CaCl_2_ stimuli regardless of Pmk1 MAPK activity. Since micafungin and CaCl_2_ are intracellular calcium inducers, our data reveal a novel role of the autophagy regulator Atg1 to induce cell death upon calcium overload independent of its role in Pmk1 MAPK activation.

## INTRODUCTION

Autophagy is a universal mechanism to maintain cellular homeostasis in eukaryotic cells through protein degradation and organelle turnover, which is considered to contribute to energy generation and cell survival [1]. However, increasing evidence suggests that autophagy also plays a key role in cell death depending on the cellular context or environmental conditions [2, 3].

We have previously shown that the deletion of the *atg1*^+^ gene, encoding a serine/threonine kinase essential for autophagy, induced phenotypes shared by the deletion mutants of the components of the Pmk1 MAPK signaling pathway, a fission yeast orthologue of the ERK MAPK pathway [4]. These phenotypes include the *vic* phenotype and the suppression of the growth inhibition induced by overexpression of Pck2, an upstream regulator of Pmk1 MAPK. The *vic* phenotype represents “viability in the presence of immunosuppressant and Cl^−^”, a shared phenotype associated with deletion or inhibition of the components of the Pmk1 MAPK signaling pathway. Deletion of the *atg1*^+^ gene also suppressed the growth inhibition associated with the overexpression of Pck2, an upstream activator of Pmk1. It should be mentioned that the degree of the *vic* phenotype observed in the Δ*atg1* mutant is weaker than that of the Δ*pmk1* mutant [4]. These data suggested that although Atg1 functions in the Pmk1 signaling pathway, the contribution may be weaker than that of MAPKK or MAPKKK.

Here, we conducted biochemical analyses and obtained data consistent with the role of Atg1 in promoting Pmk1 MAPK signaling activation, including the impact of the deletion and overexpression of *atg1*^+^ on Pmk1 phosphorylation levels. Unexpectedly, overexpression of *atg1*^+^ induces cell death, which cannot be suppressed by Pmk1 deletion. Furthermore, Δ*atg1* mutants display tolerance to Ca^2+^-increasing stimuli, micafungin, and Ca^2+^, in contrast to the severe sensitivity observed in Δ*pmk1.* Overexpression of Atg1 confers cell death both in the WT and Pmk1 deletion background, indicating that Atg1 and Pmk1 do not function in a linear fashion in cell death. Our findings reveal additional roles of Atg1 in mediating the cell death response upon calcium overload in the vegetative condition.

## RESULTS

### Identification of Atg1 as an activator of Pmk1 MAPK signaling

**Figure 1:**
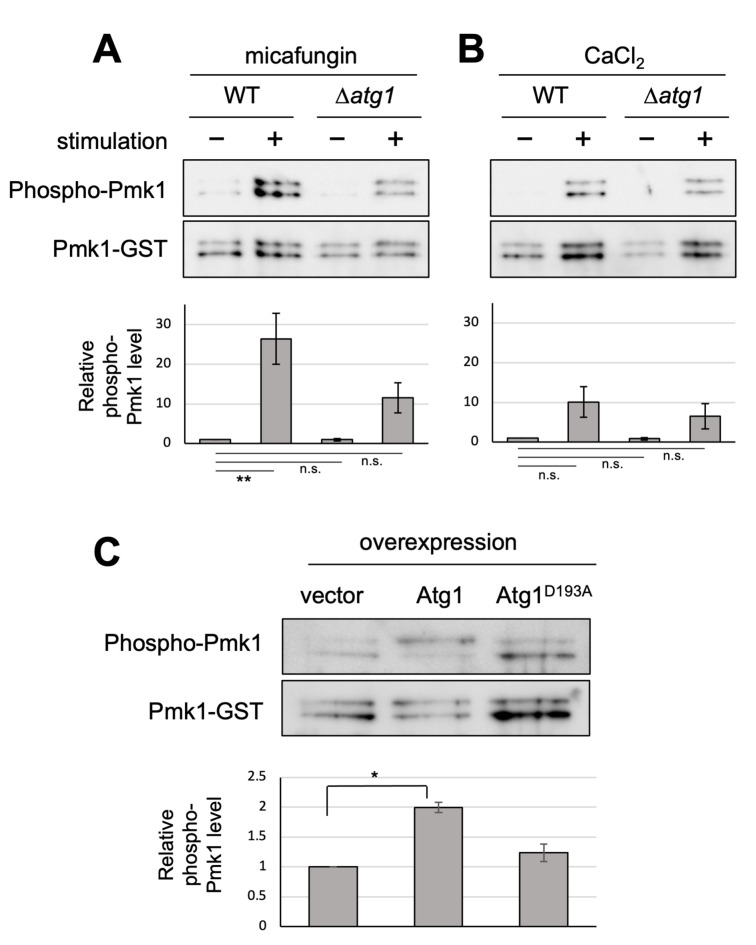
FIGURE 1: Atg1 facilitates the phosphorylation of Pmk1. (**A and B**) Impact of the deletion of *atg1^+^* on Pmk1 phosphorylation. Wild-type (WT) and Δ*atg1* cells expressing the C-terminal GST-tagged Pmk1 from the endogenous *pmk1* promoter were grown in EMM supplemented with 2 µg/ml micafungin (A) or with 200 mM CaCl2 (B) for 60 min at 27°C. Cell lysates bound to glutathione beads were immunoblotted with anti-phospho-ERK antibodies and anti-GST antibodies to detect phosphorylated Pmk1 and Pmk1-GST (loading control), respectively. Upper panel: representative immunoblot. Lower panel: Relative quantification of phosphorylated Pmk1 normalized by Pmk1-GST. Bar graphs show relative values to WT cells grown in the absence of micafungin nor CaCl2 as the mean ± standard error of the mean (SEM) of five independent experiments. N = 5; **p < 0.01 as assessed by a one-way ANOVA followed by the Dunnett's test for multiple comparisons. n.s. not significant. (**C**) Impact of overexpression of *atg1^+^* on the Pmk1 phosphorylation. Δ*atg1* cells expressing Pmk1-GST under the endogenous *pmk1* promoter and harboring pREP1-GFP, pREP1-*atg1^+^*-GFP, or pREP1-*atg1D^193A^*-GFP were grown in EMM without thiamine for 20 hr at 27°C. Cell lysates bound to glutathione beads were immunoblotted with anti-phospho-Pmk1 and anti-GST antibodies and quantified as described in (B). Bar graphs represent the mean ± SEM (N = 3; *p < 0.05 as assessed by Welch's two sample *t*-test). The levels of overexpression of WT Atg1 and kinase dead Atg1 were approximately equal (Figure S1).

Our previous genetic screen revealed that the deletion of the *atg1*^*+*^ gene displayed the *vic* phenotype [4]. Here, we further characterized Δ*atg1* mutants to obtain clues regarding the roles of Atg1 in the vegetative stage. To investigate if Atg1 can affect Pmk1 MAPK signaling activation, Pmk1 MAPK phosphorylation levels were investigated in *atg1* deletion cells in comparison with the WT cells. The basal Pmk1 phosphorylation status was not significantly affected in the *atg1* deletion cells (**Figure 1A**). We then treated cells with micafungin, a non-competitive inhibitor of 1,3-β-glucan synthase, which has been reported to stimulate cell integrity MAPK signaling [5]. Micafungin treatment significantly increased Pmk1 phosphorylation levels in the WT cells, and this induction of Pmk1 MAPK activation was impaired in *atg1* deletion cells (**Figure 1A**). Since micafungin was shown to induce intracellular Ca^2+^ concentrations [6, 7], we next investigated if the addition of CaCl_2_ can affect Pmk1 MAPK activation. 0.2 M CaCl_2_ also stimulated Pmk1 MAPK activation, and the deletion of *atg1* impaired the rise in the Pmk1 phosphorylation (**Figure 1B**). Thus, Atg1 may be required for facilitating MAPK activation in response to cell integrity-damaging stimuli and calcium stimuli.

We next examined the effect of the overexpression of Atg1. The WT cells overexpressing Atg1 displayed significantly higher levels of Pmk1 MAPK phosphorylation, in the absence of any stimuli, as compared with the cells harboring the control vector alone (**Figure 1C**). Notably, overexpression of Atg1^D193A^, the kinase-dead mutant of Atg1 [8], did not induce Pmk1 activation (**Figure 1C**), indicating that this induction of Pmk1 MAPK activation is dependent on the kinase activity of Atg1. We confirmed that the levels of overexpression of the WT and kinase-dead Atg1^D193A^ are approximately equal (Figure S1), indicating that the differences in the levels of Pmk1 activation associated with the overexpression of the WT Atg1 or the kinase-dead Atg1^D193A^ are derived from the Atg1 kinase activity. Thus, these data are consistent with the genetic interaction that Atg1 acts upstream of Pmk1 MAPK to promote MAPK activation.

### The role of Atg1 as a mediator of cell death in response to calcium overload

**Figure 2:**
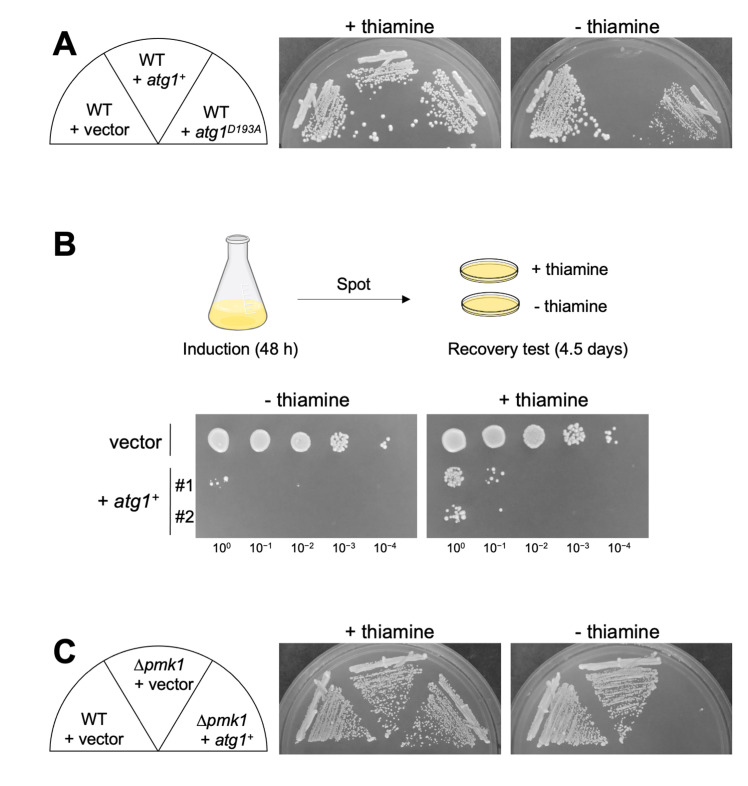
FIGURE 2: Atg1 influences the cell viability independently on the Pmk1 MAPK pathway. (**A**) Influences of overexpression of Atg1 and the kinase-defective variant Atg1^D193A^ on cell growth. Cells transformed with the thiamine repressible-expression vectors, pREP1-GFP, pREP1-*atg1^+^*-GFP, or pREP1-*atg1^D193A^*-GFP were streaked onto an EMM plate with or without 4 µM thiamine and incubated for 5 days at 27°C. (**B**) Measurement of the viability of the cells subjected to Atg1 overexpression. Cells transformed with pREP1-*atg1^+^*-GFP or its control vector were incubated in liquid EMM in the absence of thiamine for 48 hours at 27°C. The cells were then 10-fold serially diluted as indicated (10^0^ to 10^−4^) starting from OD_660_ = 0.5, and 5 µL were spotted onto EMM plates with or without thiamine. Plates were incubated for 4.5 days at 27°C. Two independent transformant clones (#1 and #2) for pREP1-*atg1^+^*-GFP were tested. (**C**) Overexpression of Atg1 induces cytotoxicity independently on Pmk1. Δ*pmk1* cells harboring pREP1-GFP or pREP1-*atg1^+^*-GFP and wild-type cells harboring pREP1-GFP were streaked onto an EMM plate with or without 4 µM thiamine and incubated for 4 days at 27°C.

We further investigated the impact of Atg1 overexpression on cell growth. Since the hyperactivation of Pmk1 MAPK signaling leads to cell growth inhibition [9], Atg1 overexpression may exert the same effect by promoting Pmk1 MAPK signaling activation. The growth of the WT cells expressing *atg1*^+^ was much slower than that of the WT cells harboring the control vector alone, indicating that Atg1 induces a reduction of cell growth when overproduced (**Figure 2A**). The growth reduction was also observed when the kinase-inactivating *atg1*^D193A^ was overexpressed, although the effect was modest as compared with that induced by the WT *atg1*^+^. To determine whether or not cell death is occurring or if growth is simply inhibited upon Atg1 overexpression, we measured colony-forming units (CFUs) by spotting cells on the EMM plates with or without thiamine after 48 hours of induction of Atg1 overexpression (**Figure 2B**). The results showed that the viability of the cells subjected to Atg1 overexpression is quite low even when plated in the repressed condition, showing approximately less than 1% of cell viability as compared with that of the cells harboring the control vector alone. Thus, the reduction of cell growth induced by Atg1 overexpression was irreversible, indicating that Atg1 overexpression causes cell death rather than growth inhibition. Notably, the *pmk1* deletion did not suppress the cell death induced by Atg1 overexpression, indicating that the Atg1-mediated cell death does not require Pmk1 MAPK activation (**Figure 2C**).

To further obtain clues regarding the Atg1-mediated cell death, we investigated if cell death by Atg1 overexpression can be suppressed by the deletion of other Atg genes. If the deletion of Atg genes may affect the toxicity induced by Atg1 overexpression, the downstream of cell death by Atg1 overexpression is not Pmk1, but the Atg pathway. Among the strains deleted for each of the 13 Atg genes that we examined, none of them suppressed the Atg1 overexpression-induced cell death (Figure S2). We, therefore, consider that the Atg pathway, as a whole, is not required for the execution of cell death triggered by Atg1.

**Figure 3:**
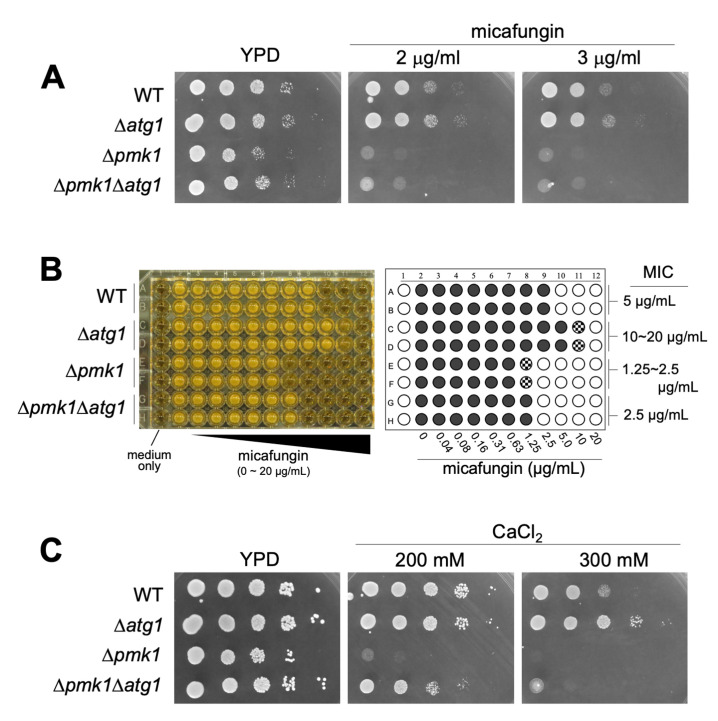
FIGURE 3: Δ*atg1* cells are resistant to micafungin and calcium. (**A**) Cells as indicated were serially diluted 10^0^, 10^−1^, 10^−2^, 10^−3^, 10^−4^ and 5 µl were spotted onto YPD plates containing the indicated concentrations of micafungin. Plates were incubated at 27°C for 2 days. (**B**) Minimum Inhibitory Concentration (MIC) of micafungin. Cells as indicated were grown to mid-log phase and adjusted to OD_660_ = 0.5. The cells were then diluted 300-fold with fresh liquid YES and incubated in a 96-well plate with the indicated concentrations of micafungin for 2.5 days at 27°C. The most left wells contain medium only (sterility control). Right panel is a schematic illustration of the result of MIC assay. Open circle (○), closed circle (•), and shaded circle indicate no growth, full growth and a slight growth, respectively. (**C**) Cells as indicated were serially diluted 10^0^, 10^−1^, 10^−2^, 10^−3^, 10^−4^ and 5 µL were spotted onto YPD plates containing the indicated concentrations of CaCl_2_. Plates were incubated at 27°C for 3 days.

Next, we investigated the phenotypic consequence of the Atg1 deletion on micafungin sensitivity as the loss-of-function mutants or deletion of the components of the Pmk1 MAPK signaling pathway shared the phenotype of micafungin hypersensitivity. These components include Pmk1, Pek1 MAPKK, Mkh1 MAPKKK, Pck2, Rho2, Cpp1 farnesyl transferase, and Cwg2 geranylgeranyl transferase [9–11]. Surprisingly, *atg1* deletion cells displayed tolerance to micafungin, in contrast to the severe sensitivity to the compound associated with Δ*pmk1*. As shown in **Figure 3A**, the growth of Δ*atg1* was better than that of the WT cells in the presence of micafungin, whereas the growth of Δ*pmk1* was severely inhibited by the compound. Thus, although genetic and biochemical experiments suggest that Atg1 acts upstream of Pmk1 MAPK signaling, *atg1* deletion did not induce micafungin hypersensitivity but rather enhanced tolerance to micafungin. To further characterize the phenotypic discrepancies between Δ*atg1* and Δ*pmk1* cells in terms of micafungin tolerance, we next analyzed the effect of the *pmk1* deletion on micafungin resistance elicited by *atg1* deletion. The Δ*atg1*Δ*pmk1* double mutant cells displayed an intermediate sensitivity to micafungin as the double mutants grew faster than Δ*pmk1* cells and slower than Δ*atg1* cells in the medium containing micafungin (**Figure 3A**).

To evaluate the difference in the micafungin sensitivities between the single and the double mutant strains more quantitatively, we analyzed the growth of each strain against a wide range of concentrations of micafungin using a 96-well plate format with a liquid medium and determined the Minimum Inhibitory Concentration (MIC) of micafungin in each strain. As shown in **Figure 3B**, the MIC in Δ*atg1* cells is 10∼20 μg/ml, apparently higher than that in the WT cells (5 μg/ml). Moreover, the MIC in Δ*pmk1* cells (1.25∼2.5 μg/ml) is lower than that in the WT cells and higher than that in the Δ*pmk1*Δ*atg1* double mutant strain (2.5 μg/ml). Thus, *pmk1* deletion partly suppressed micafungin resistance associated with *atg1* deletion, suggesting that the tolerance of *atg1* deletion to the growth inhibition by micafungin is mediated not only by Pmk1 signaling but also by additional signaling mechanism(s). To further analyze the phenotypes of Δ*atg1*, we investigated the effect of the *atg1* deletion on the sensitivity to calcium, which also stimulates Pmk1 activation. Again, the growth of Δ*atg1* was better than that of the WT cells in the YPD medium containing 0.2 M CaCl_2_, whereas the growth of Δ*pmk1* was severely inhibited in the same medium (**Figure 3C**). The Δ*atg1*Δ*pmk1* double mutant displayed an intermediate sensitivity to CaCl_2_ (**Figure 3C**), again showing that the suppression by *pmk1* deletion is partial. We also examined the CFU assay using the YES medium to evaluate the micafungin and CaCl_2_ sensitivities and obtained similar results to that obtained with the YPD medium (Figure S3). Thus, Atg1 functions to hamper cell growth in the presence of CaCl_2_ and micafungin regardless of Pmk1 MAPK activity.

## DISCUSSION

Here, we provide evidence that the fission yeast Atg1 regulates cell death responses upon intracellular calcium load in addition to its role in promoting Pmk1 MAPK.

First, Atg1 was shown to positively regulate Pmk1 MAPK activation based on the findings that Atg1 deletion inhibits Pmk1 MAPK phosphorylation upon micafungin and CaCl_2_ stimuli and that Atg1 overproduction stimulates Pmk1 phosphorylation. These data are consistent with our previous genetic data showing that *atg1* deletion phenotypically recapitulates deletion mutants of the upstream components of the Pmk1 MAPK pathway [4]. In mammals, Martinez-Lopez *et al.* proposed an unconventional function of ATG proteins in the regulation of ERK phosphorylation by showing that deleting *Atg7* or *Atg5* or blocking LC3 lipidation decreases ERK phosphorylation [12]. Intriguingly, components of the ERK signaling cascade are associated with autophagosomes, proposing that autophagosomes serve as scaffolds or cellular platforms regulating ERK phosphorylation [12, 13]. Our previous study also showed the involvement of the autophagy system as a whole in the regulation of MAPK, because some autophagy-related gene deletions phenotypically recapitulate MAPK signaling inhibition [4]. It is noteworthy that Atg1 overexpression promotes Pmk1 signaling in a kinase-dependent manner. It has been reported that Atg1 kinase organizes autophagosome formation by phosphorylating Atg9 in budding yeast [14]. Thus, the concept of autophagosome formation initiated by Atg1 as a mechanism to regulate ERK phosphorylation via spatial coordination of ERK signaling may be universal. Whether other autophagy-related genes also facilitate Pmk1 MAPK activation, or Atg1 specifically functions to initiate MAPK signaling stimulation via its kinase activity would be an important issue for future study.

Secondly, we characterized the cell death phenotypes mediated by Atg1. Cell death stimulation was observed when Atg1 is overexpressed in WT cells. The failure of *pmk1* deletion to suppress the cell death associated with the overexpression of Atg1 implicates that the Atg1-mediated cell death response is not solely explained by Pmk1 MAPK activation. Further phenotypic analysis showed that *atg1* deletion induced tolerance to micafungin and CaCl_2_ in contrast to the severe sensitivities of *pmk1* deletion to these agents. Micafungin and CaCl_2_ shared the property to stimulate intracellular calcium concentrations and calcium signaling [6]. Thus, Atg1 plays a key role to mediate cell death in response to the two calcium-stimulating stresses, whereas Pmk1 is required for proliferation during the same stresses. Thus, it seems plausible to hypothesize that the cell death system involving Atg1 is executed in cells with maladaptation to calcium-increasing stimuli. Importantly, the cell death execution mediated by Atg1 was observed both in the WT and *pmk1* deletion background, again suggesting that Atg1 mediates cell death independently of Pmk1 MAPK activity. It should be noted that Atg1 overexpression confers toxicity even in cells deleted for each of the Atg pathway genes. These results suggest that neither Pmk1 nor the Atg pathway serves as a downstream target of Atg1-mediated cell death. We then investigated if the calcium tolerance phenotype associated with the *atg1* deletion is shared by other Atg pathway deletion cells. The results showed that many of the genes deleted for the Atg pathway exhibited similar levels of calcium tolerance to that of the *atg1* deletion cells (Figure S4). These results suggest that not only Atg1 but also the Atg pathway at large seems to be involved in the calcium-related cell death process. However, this will not exclude the possibility that Atg1 overexpression induces some proteostasis stress responses which may trigger cell death irrelevant to the Atg pathway. Intriguingly, caspofungin, another candin fungicidal compound that also inhibits 1,3-β-D-glucan synthase, has been reported to induce programmed cell death in *Saccharomyces cerevisiae* and *Candida albicans* [15, 16]. In addition, micafungin induced apoptosis in *Candida parapsilosis* [17]. Therefore, the cell death response elicited by micafungin may be executed by apoptosis machinery in fission yeast. Micafungin and other echinocandin class of antifungal drugs have assumed an increasingly vital role in the therapy of invasive candidiasis [18]. Although autophagy often accompanies cell death following various toxic insults, autophagy-dependent cell death is highly contextual [19]. In this regard, elucidating the cell death mechanism mediated by Atg1 in response to micafungin may contribute to proposing a novel strategy for anti-fungal treatment.

## MATERIALS AND METHODS

### Yeast strains, media, and molecular biology

*Schizosaccharomyces pombe* strains and plasmids used in this study are listed in Table 1. The complete medium YPD or YES, and the minimal medium EMM have been described previously [20, 24]. Standard yeast culture and genetic methods were used except where noted [21, 22]. Kinase-defective variant of Atg1 (Atg1^D193A^) was generated by inverse PCR using Vent DNA Polymerase (NEB) with the sense primer (#2144) 5'-TAGCAGCCTTTGGTTTTGCACGTTACCTTCAAACGTC-3' and the reverse primer (#2130) 5'-GGTAACGTGCAAAACCAAAGGCTGCTAATTTAAGCAT-3'.

**Table 1.** Strains and plasmids used in this study.

**Strain**	**Genotype**	**Reference**
HM123	*h^−^ leu1-32*	Lab stock
SP341	*h^−^ leu1-32 ura4-D18 atg1::ura4^+^*	[24]
SP385	*h^−^ leu1-32 ura4-D18 pmk1::ura4^+^*	[25]
KP2178	*h^−^ leu1-32 pmk1*::*KanMX6*	Lab stock
SP2857	*h^−^ leu1-32 ura4-D18 atg1::ura4^+^ pmk1*::*KanMX6*	[4]
SP2231	*h^−^ leu1-32 pmk1^+^-GST*::*KanMX6*	[25]
SK2	*h^−^ leu1-32 ura4-C190T atg2::ura4* ^+^	[26]
SK3	*h^−^ leu1-32 ura4-C190T atg3::ura4* ^+^	[26]
SK4	*h^−^ leu1-32 ura4-C190T atg4::ura4* ^+^	[26]
SK5	*h^−^ leu1-32 ura4-C190T atg5::ura4* ^+^	[26]
SK6	*h^−^ leu1-32 ura4-C190T atg6::ura4* ^+^	[26]
SK7	*h^−^ leu1-32 ura4-C190T atg7::ura4* ^+^	[26]
SK8	*h^−^ leu1-32 ura4-C190T atg8::ura4* ^+^	[26]
SK9	*h^−^ leu1-32 ura4-C190T atg9::ura4* ^+^	[26]
SK10	*h^−^ leu1-32 ura4-C190T atg12::ura4* ^+^	[26]
SK11	*h^−^ leu1-32 ura4-C190T atg13::ura4* ^+^	[26]
SK12	*h^−^ leu1-32 ura4-C190T atg15::ura4* ^+^	[26]
SK13	*h^−^-leu1-32 ura4-C190T atg17::ura4* ^+^	[26]
SK14	*h^−^ leu1-32 ura4-C190T atg22::ura4* ^+^	[26]

**Plasmid**	**Description**	**Reference**
pKB1037	LEU2 marker multi-copy vector	[27]
pKB2728	pREP1-GFP	Lab stock
pKD3327	pREP1-*atg1^+^*-GFP	This study
pKD4310	pREP1-*atg1^D193A^*-GFP	This study

### Purification and Detection of phosphorylated Pmk1

Cells expressing GST-tagged Pmk1 from the endogenous *pmk1*^*+*^ promoter were suspended in ice cold lysis buffer [1% Triton X, 30 mM Tris-HCl (pH 8.0), 2 mM EDTA, 0.1% 2-mercaptoethanol plus protease inhibitor mixtures and phosphatase inhibitor mixtures] and homogenized with glass beads using Multi-beads Shocker (Yasui Kikai, Osaka, Japan). The lysates were cleared by centrifugation at 15,000 rpm for 15 min, and GST-tagged Pmk1 was purified by glutathione agarose beads. The purified proteins were resolved in 10% SDS-PAGE gels and transferred to the PVDF membrane (Millipore). Phosphorylated GST-Pmk1 was immunoreacted with either rabbit anti-phospho-p42/44 antibodies (Cell Signaling) or rabbit anti-phospho-Pmk1 antibodies [23] and anti-rabbit-IgG HRP-conjugated secondary antibody (Cell Signaling), then developed with Chemi-Lumi One Super (Nacalai tesque, Japan). The blots were stripped using WB stripping solution (Nacalai tesque) and reprobed with rabbit anti-GST antibodies [23] to measure the amount of total GST-Pmk1 loaded in each lane. Protein levels were quantified using MULTI GAUGE Ver. 3.2 software (Fujifilm, Japan)

### MIC assay

Strains were grown to mid-log phase in liquid YES at 27°C and adjusted to OD_660_ = 0.5, and then diluted 150-fold with the fresh medium. 100 µl of aliquots were pipetted into a 96-well plate and mixed with the equal amout of YES medium containing the different concentrations (2-fold serial dilutions) of micafungin. Plates were incubated at 27°C for 3 days.

### Statistical analysis

Data are expressed as means ± standard error of the mean (SEM). We have run a one-way ANOVA, followed by a post hoc test using Dunnett's test for multiple comparisons. The Welch's two sample *t*-test was used to determine the level of significance of differences between the two groups. Statistical significance was defined as *p*<0.05.

## SUPPLEMENTAL MATERIAL

Click here for supplemental data file.

All supplemental data for this article are available online at www.microbialcell.com/researcharticles/2023a-takasaki-microbial-cell/.

## Abbreviations:

WT: – wild-type,
vic: – viability in the presence of immunosuppressant and Cl^−^,
CFUs: – colony-forming units,
MIC: – Minimum Inhibitory Concentration.

## References

[1] Mizushima N, Komatsu M (2011). Autophagy: Renovation of Cells and Tissues.. Cell..

[2] Gozuacik D, Kimchi A (2007). Autophagy and Cell Death.. Curr Top Dev Biol..

[3] Doherty J, Baehrecke EH (2018). Life, death and autophagy.. Nat Cell Biol..

[4] Takasaki T, Utsumi R, Shimada E, Tomimoto N, Satoh R, Sugiura R (2022). Autophagy-related genes genetically interact with Pmk1 MAPK signaling in fission yeast.. Micropublication Biology..

[5] Takada H, Nishimura M, Asayama Y, Mannse Y, Ishiwata S, Kita A, Doi A, Nishida A, Kai N, Moriuchi S, Tohda H, Giga-Hama Y, Kuno T, Sugiura R (2007). Atf1 is a target of the mitogen-activated protein kinase Pmk1 and regulates cell integrity in fission yeast.. Molecular biology of the cell..

[6] Deng L, Sugiura R, Takeuchi M, Suzuki M, Ebina H, Takami T, Koike A, Iba S, Kuno T (2006). Real-Time Monitoring of Calcineurin Activity in Living Cells: Evidence for Two Distinct Ca2+-dependent Pathways in Fission Yeast.. Mol Biol Cell..

[7] Ogata F, Satoh R, Kita A, Sugiura R, Kawasaki N (2017). Evaluation of a novel method for measurement of intracellular calcium ion concentration in fission yeast.. J Toxicol Sci..

[8] Pan Z-Q, Shao G-C, Liu X-M, Chen Q, Dong M-Q, Du L-L (2020). Atg1 kinase in fission yeast is activated by Atg11-mediated dimerization and cis-autophosphorylation.. Elife..

[9] Ma Y, Kuno T, Kita A, Asayama Y, Sugiura R (2006). Rho2 Is a Target of the Farnesyltransferase Cpp1 and Acts Upstream of Pmk1 Mitogen-activated Protein Kinase Signaling in Fission Yeast.. Mol Biol Cell..

[10] Doi A, Kita A, Kanda Y, Uno T, Asami K, Satoh R, Nakano K, Sugiura R (2015). Geranylgeranyltransferase Cwg2-Rho4/Rho5 module is implicated in the Pmk1 MAP kinase-mediated cell wall integrity pathway in fission yeast.. Genes Cells..

[11] Ma Y, Kuno T, Kita A, Nabata T, Uno S, Sugiura R (2006). Genetic Evidence for Phospholipid-Mediated Regulation of the Rab GDP-Dissociation Inhibitor in Fission Yeast.. Genetics..

[12] Martinez-Lopez N, Athonvarangkul D, Mishall P, Sahu S, Singh R (2013). Autophagy proteins regulate ERK phosphorylation.. Nature communications..

[13] Martinez-Lopez N, Singh R (2014). ATGs: Scaffolds for MAPK/ERK signaling.. Autophagy..

[14] Papinski D, Schuschnig M, Reiter W, Wilhelm L, Barnes CA, Maiolica A, Hansmann I, Pfaffenwimmer T, Kijanska M, Stoffel I, Lee SS, Brezovich A, Lou JH, Turk BE, Aebersold R, Ammerer G, Peter M, Kraft C (2014). Early steps in autophagy depend on direct phosphorylation of Atg9 by the Atg1 kinase.. Molecular cell..

[15] Hao B, Cheng S, Clancy CJ, Nguyen MH (2013). Caspofungin Kills Candida albicans by Causing both Cellular Apoptosis and Necrosis.. Antimicrob Agents Ch..

[16] Donaghey F, Helming K, McCarthy M, Rogers S, Austriaco N (2014). Deletion of *AIF1* but not of *YCA1/MCA1* protects *Saccharomyces cerevisiae* and *Candida albicans* cells from caspofungin-induced programmed cell death.. Microb Cell..

[17] Shirazi F, Lewis RE, Kontoyiannis DP (2015). Micafungin induced apoptosis in Candida parapsilosis independent of its susceptibility to micafungin.. Microb Cell..

[18] Gonzalez-Lara M, Ostrosky-Zeichner L (2020). Invasive Candidiasis.. Semin Resp Crit Care..

[19] Denton D, Kumar S (2019). Autophagy-dependent cell death.. Cell Death Differ..

[20] Toda T, Dhut S, Superti-Furga G, Gotoh Y, Nishida E, Sugiura R, Kuno T (1996). The fission yeast pmk1+ gene encodes a novel mitogen-activated protein kinase homolog which regulates cell integrity and functions coordinately with the protein kinase C pathway.. Mol Cell Biol..

[21] Rose MD, Winston F, Hieter P (1990). Methods in yeast genetics: A laboratory course manual.. Cold Spring Harbor Laboratory Press..

[22] Alfa C, Fantes P, Hyams J, Warbrich M and E (1993). Experiments with fission yeast: A laboratory course manual.. Trends Genet..

[23] Sugiura R, Toda T, Dhut S, Shuntoh H, Kuno T, Kuno T (1999). The MAPK kinase Pek1 acts as a phosphorylation-dependent molecular switch.. Nature..

[24] Bimbó A, Jia Y, Poh SL, Karuturi RKM, Elzen N den, Peng X, Zheng L, O'Connell M, Liu ET, Balasubramanian MK, Liu J (2005). Systematic deletion analysis of fission yeast protein kinases.. Eukaryot Cell..

[25] Kanda Y, Satoh R, Takasaki T, Tomimoto N, Tsuchiya K, Tsai CA, Tanaka T, Kyomoto S, Hamada K, Fujiwara T, Sugiura R (2021). Sequestration of the PKC ortholog Pck2 in stress granules as a feedback mechanism of MAPK signaling in fission yeast.. J Cell Sci..

[26] Mukaiyama H, Kajiwara S, Hosomi A, Giga-Hama Y, Tanaka N, Nakamura T, Takegawa K (2009). Autophagy-deficient Schizosaccharomyces pombe mutants undergo partial sporulation during nitrogen starvation.. Microbiology+..

[27] Ma Y, Sugiura R, Saito M, Koike A, Sio SO, Fujita Y, Takegawa K, Kuno T (2007). Six new amino acid-auxotrophic markers for targeted gene integration and disruption in fission yeast.. Curr Genet..

