# Supplementary material for: Atg1, a key regulator of autophagy, functions to promote MAPK activation and cell death upon calcium overload in fission yeast
Source: Microb Cell. 2023 May 10;10(6):133–40. doi: 10.15698/mic2023.06.798 (PMC10236205; doi:10.15698/mic2023.06.798)
Supplement: Supplementary file 1 [file mic-10-133-s01.pdf]

Supplemental Information

Atg1, a key regulator of autophagy, functions to promote MAPK activation and cell death upon calcium overload in fission yeast

Teruaki Takasaki[1], Ryosuke Utsumi[1], Erika Shimada[1], Asuka Bamba[1], Kanako Hagihara[2], Ryosuke Satoh[1], and Reiko Sugiura[1]*

[1]Laboratory of Molecular Pharmacogenomics, Department of Pharmaceutical Sciences, Faculty of Pharmacy, Kindai University, Higashi-Osaka, 577-8502, Japan.
[2]Laboratory of Hygienic Science, Department of Pharmacy, Hyogo Medical University, Kobe, 650-8530, Japan.

*To whom correspondence should be addressed:
TEL: +81-6-4307-3642; FAX: +81-6-6730-1394, E-mail: sugiurar@phar.kindai.ac.jp

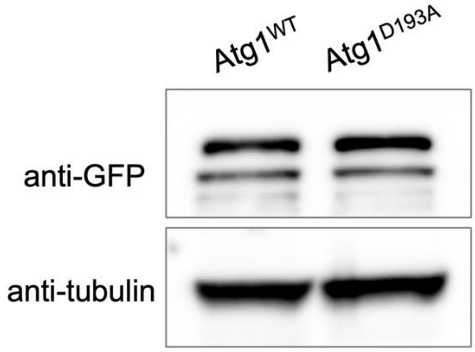

FIGURE S1: Levels of the wild-type and kinase-dead Atg1 upon overexpression. Strains expressing C-terminal GST-tagged Pmk1 under the endogenous *pmk1* promoter were transformed with either pREP1-*atg1*$^{+}$-GFP or pREP1-*atg1*^{D193A}-GFP, then grown in EMM without thiamine at 27°C. Cell lysates were immunoblotted with anti-GFP and anti-tubulin antibodies. The levels of the wild-type Atg1 and kinase-dead Atg1 were approximately equal.

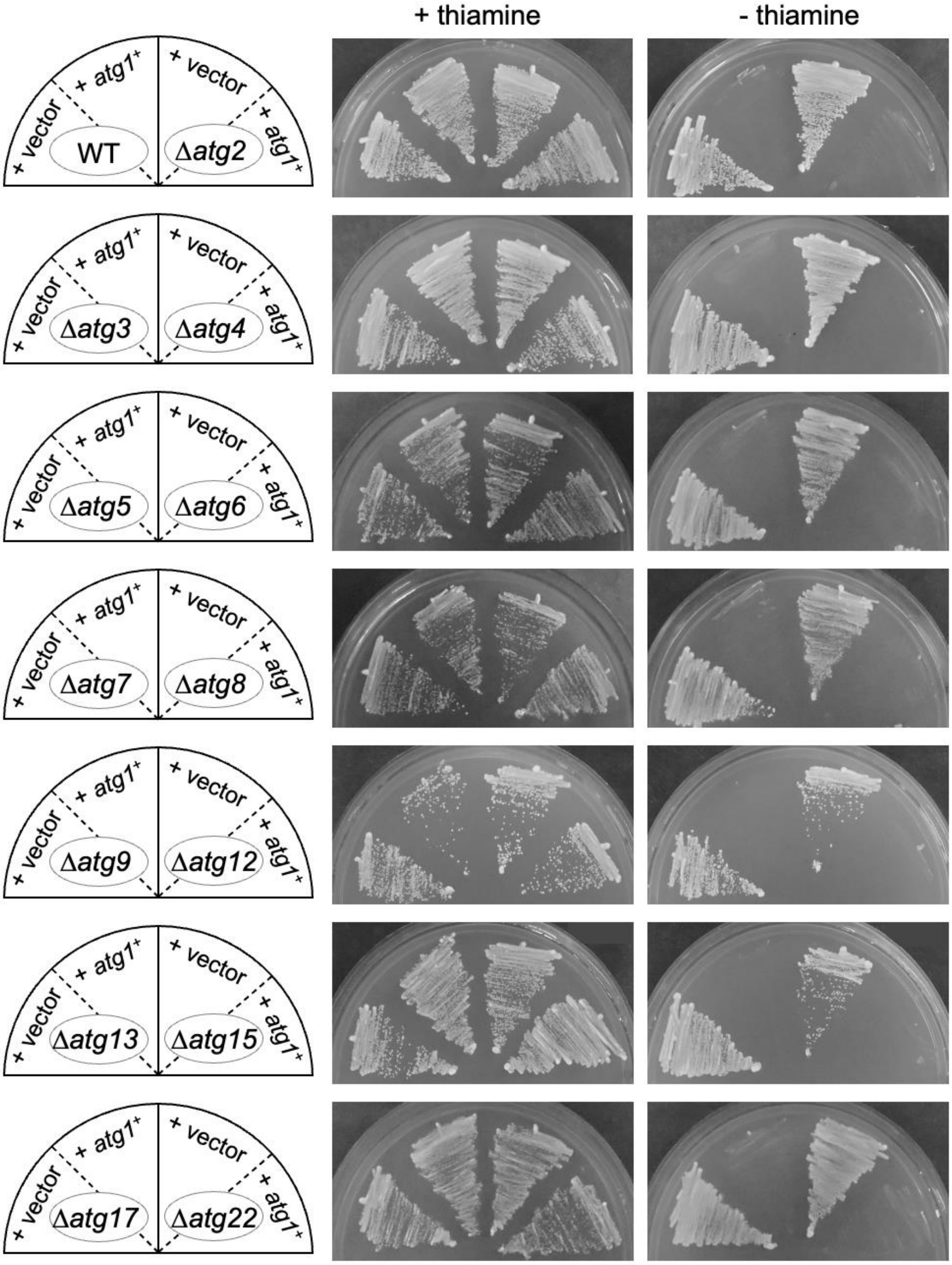

FIGURE S2: Cell death induced by overexpression of Atg1 was not suppressed by the deletion of other *atg* genes.

Strains lacking the indicated *atg* genes were transformed with pREP1-GFP vector or pREP1-*atg1*$^{+}$-GFP, streaked onto an EMM plate with or without 4 μM thiamine, and incubated for 4 days at 27˚C.

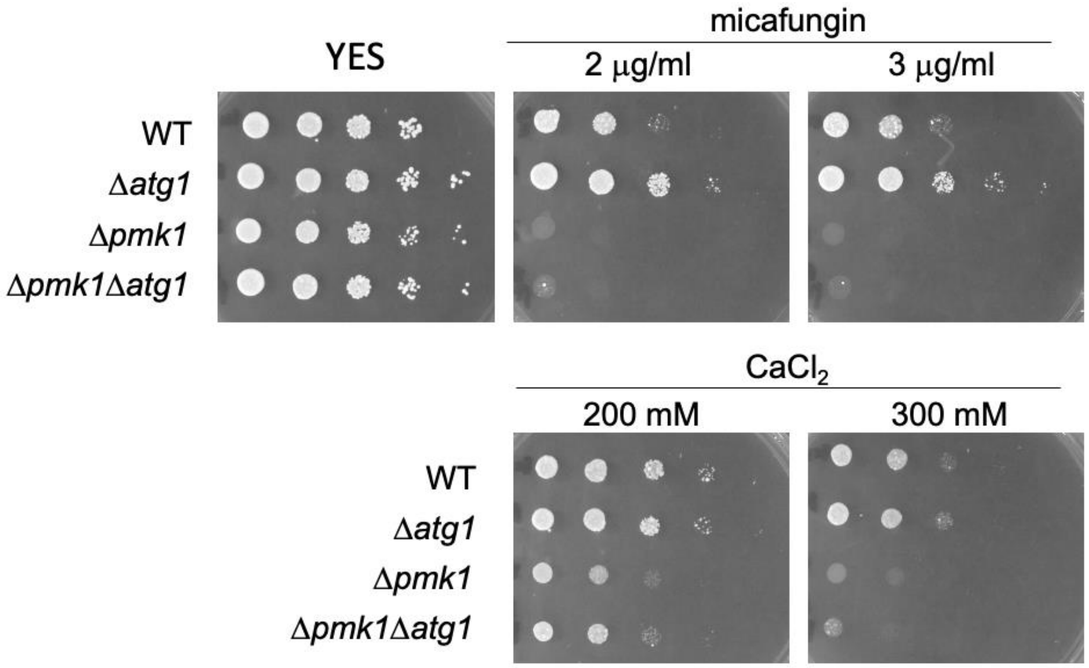

FIGURE S3: CFU assay for the micafungin and $CaCl_2$ sensitivity using YES plates. Cells as indicated were serially diluted 10^0, 10^{-1}, 10^{-2}, 10^{-3}, 10^{-4} (starting from OD_{660} = 0.5) and 5 μL were spotted onto YES plates containing the indicated concentrations of micafungin or $CaCl_2$. Plates were incubated at 27°C for 2 ~ 3 days.

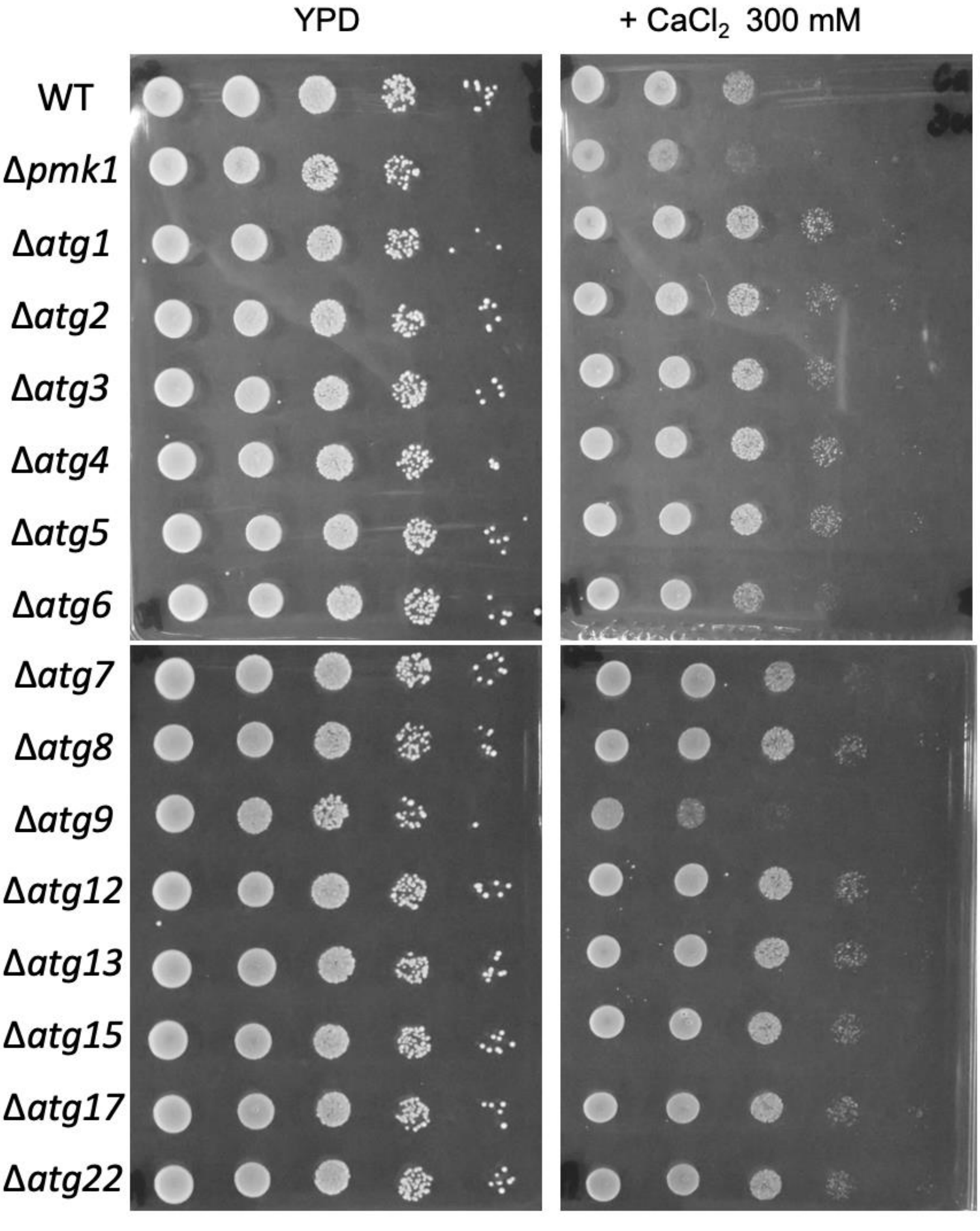

FIGURE S4: Calcium tolerance of strains lacking *atg* genes.

Cells as indicated were serially diluted 10^0, 10^{-1}, 10^{-2}, 10^{-3}, 10^{-4} (starting from OD_{660} = 0.5) and 5 μL were spotted onto YPD plates containing 300 mM $CaCl_2$. Plates were incubated at 27˚C for 3 days.
